# Association of albumin-bilirubin grade with short- and long-term mortality in patients with heart failure: a cohort study using restricted cubic splines and propensity score matching

**DOI:** 10.1186/s12872-025-04760-2

**Published:** 2025-04-23

**Authors:** Xiaopo Gao, Cheng Li, Yurou Wang, Yun Cu, Yingfang Zheng, Hongkai Dai, Xinrun Yuan, Jinlong Luo, Chengye Zhan

**Affiliations:** 1 Department of Emergency Medicine, Tongji Hospital, Tongji Medical College, Huazhong University of Science and Technology, Wuhan, China; 2 Department of Critical Care Medicine, Tongji Hospital, Tongji Medical College, Huazhong University of Science and Technology, Wuhan, China

**Keywords:** Heart failure, Prognosis, The albumin-bilirubin grade

## Abstract

**Objective:**

Heart failure (HF) is a chronic progressive syndrome caused by a variety of cardiovascular diseases and is associated with high morbidity, mortality, and healthcare burden. Forecasting the prognosis of HF patients at an early stage is important. Therefore, our objective was to explore the relationship between HF patients’ prognosis and the albumin-bilirubin (ALBI) grade.

**Methods:**

Data for the study were obtained from the MIMIC database. Patients with ALBI grade 1 were matched to patients with ALBI grades 2 and 3 using propensity score matching (PSM). Post-matching analyses were performed using Cox proportional hazards models, Kaplan-Meier survival analysis, restricted cubic splines (RCS), and subgroup analyses.

**Results:**

RCS analyses revealed a nonlinear relationship between ALBI grade and 30-, 90-, and 360-day mortality in patients with HF, with a threshold value identified at -1.92. When ALBI scores were below − 1.92, the risk of mortality in HF patients remained relatively stable. In contrast, as ALBI scores approached and exceeded − 1.92, the mortality risk increased rapidly. Before PSM, both ALBI grades 2 and 3 were independent predictors of mortality in patients with HF; however, after PSM, ALBI grade 2 was not statistically associated with patient mortality. This result was supported by Kaplan-Meier (K-M) analysis.

**Supplementary Information:**

The online version contains supplementary material available at 10.1186/s12872-025-04760-2.

## Introduce

Heart failure (HF) is a syndrome caused by a dysfunction of the heart’s pumping function, resulting in an inability to supply the basic metabolic needs of the whole body [1]. With an aging global population and high prevalence of chronic diseases, the incidence of HF has increased significantly and has become a major global public health challenge [2]. Patients with HF are often hospitalized because of the complexity of their condition, the high mortality rate, and the high consumption of healthcare resources [3].

In recent years, the importance of liver function in patients with HF has been gradually recognized [4]. As a key organ for metabolism and detoxification, the functional status of the liver is closely related to the severity of HF, systemic inflammation, and multi-organ dysfunction [5]. HF is often complicated by abnormal liver function, especially during the acute phase of HF, and may be associated with a worse prognosis [6]. Therefore, assessing liver function is important in determining the prognosis of patients with HF [7]. Several previous studies have shown that indicators related to liver function, such as total bilirubin, albumin, and aspartate aminotransferase, can be predictive of prognosis in patients with HF. However, a single indicator is influenced by multiple factors that affect its predictive accuracy [8–11].

The ALBI grade was often used to evaluate liver disease and has been gradually applied to the prognosis of other diseases in recent years due to its simplicity and objectivity in reflecting the comprehensive status of liver function [12, 13]. The ALBI score is calculated from albumin and total bilirubin, which is effective in differentiating between patients with different hepatic function states and has a high prognostic power [12, 14]. In addition, the ALBI grade has excellent value in predicting the prognosis of sepsis, acute kidney injury, esophageal cancer, and other diseases [15–17]. However, the association of ALBI grade with outcome in patients with HF has not been adequately studied [18].

Therefore, we wanted to explore the prognostic value in assessing the ALBI grade in patients with HF.

## Materials and methods

### Date sources

The data for this study were extracted from the MIMIC-IV database, a publicly available and rigorously checked database of intensive care unit (ICU) patients. Specifically, the MIMIC-IV database contains detailed clinical data from more than 700,000 ICU admissions from the 2008–2019 period.

Before the research team used the MIMIC-IV database, the first author of this paper, Gao, had completed the necessary CITI training (Collaborative Institutional Training Initiative) and obtained the appropriate data access (ID: 66236335). The use of the database was strictly adhered to by the Ethics Committee to ensure legal compliance in the use of the data.

### Study population and data extraction

**Fig. 1:**
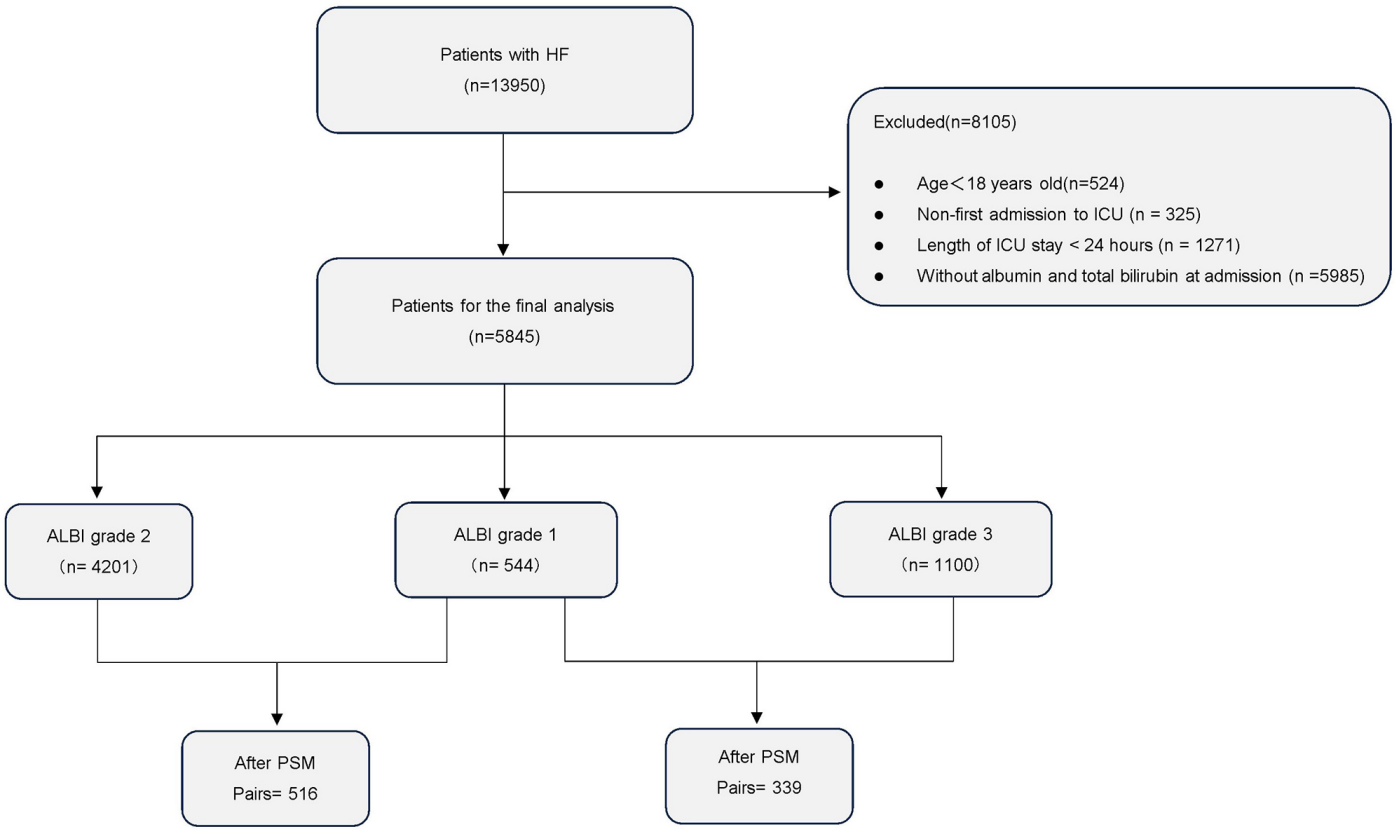
Flowchart for the inclusion of patients with heart failure

Adult patients with heart failure treated in the ICU were included in this study through the International Classification of Diseases (ICD) (See Supplementary Material 5). Exclusion criteria were (1) age < 18 years old, (2) non-first admission to ICU, (3) lack of albumin and total bilirubin data on admission, and (4) ICU stay of less than 24 h. A final total of 5845 patients were included (Fig. 1).

Our study included only patients admitted to the intensive care unit and did not cover the entire course of the disease. Albumin and bilirubin levels recorded at the time of admission to the ICU were used for calculation. ALBI was calculated using the formula ((log10 bilirubin (µmol/L) × 0.66) + (albumin (g/L) × -0.085) [12]. Meanwhile, there are three grades of ALBI: 1, 2, and 3, corresponding to ≤-2.60, >-2.60 - ≤-1.39, and >-1.39, respectively. Grade 1 indicates that the patient’s hepatic function is mildly impaired, grade 2 is moderately impaired, and grade 3 is severely impaired. Usually, the higher the grade, the worse the prognosis of the patient [12]. In addition, severe hepatic dysfunction in comorbidities is defined through a combination of ICD codes and the Model for End-Stage Liver Disease (MELD) score. MELD = 3.78×ln (Total Bilirubin) + 11.2×ln (INR) + 9.57×ln (Creatinine) + 6.4. The mortality rate of patients was significantly higher when MELD ≥ 30; therefore, in this study, severe hepatic dysfunction was defined as MELD ≥ 30 [19].

Relevant characteristics of the patients were extracted from the database: (1) demographic characteristics, including age and gender; (2) intervention; (3) comorbidities; and (4) SOFA scores; see Table 1 for details.

Variables with missing values less than 25% were retained. The missing values of glucose (62.17%), calcium (44.84%), left ventricular ejection fraction (LVEF, 85%) and creatine kinase (50.04%) were high and therefore not included in the study. The corresponding interpolation strategy was applied according to the characteristics of the variables: for variables obeying a normal distribution, the missing values were replaced by the mean, and for variables not obeying a normal distribution, the missing values were replaced by the median. This approach ensures the stability of the data set and improves the reliability of the subsequent analysis.

### Outcomes

The primary outcome of this study was 30-day all-cause mortality in patients with heart failure, and the secondary outcomes were 90- and 360-day all-cause mortality.

### Statistical analysis

Normality of continuous variables was initially assessed using the Kolmogorov-Smirnov (K-S) test. Since none of the continuous variables in this study followed a normal distribution, the median (with interquartile ranges) was used for statistical analysis. Categorical variables were presented as percentages. Based on the sample size and subgroups, continuous variables were assessed for between-group differences using the Kruskal-Wallis test, while categorical variables were compared using the chi-square test.

Cox models were employed to determine whether the ALBI score was an independent risk factor for patients with HF. First, univariate Cox regression was used to analyze baseline characteristics. Following univariate analysis, variables having a P-value < 0.05 were included in multivariate Cox regression models. Four distinct models were developed: Model 1, Model 2, Model 3 and Model 4, each containing different variables or adjustments (refer to Supplementary Material 4 for details). The association between mortality in HF patients and various ALBI grades was evaluated using K-M curves. Additionally, subgroup analyses were performed to investigate the relationship between ALBI grade and prognosis in different subgroups of patients. Restrictive cubic splines (RCS) were employed to assess the nonlinear relationship between ALBI score and patient mortality. We also evaluated the interaction between ALBI grade and the variables in Model 3 to determine whether these factors change the prognostic value of ALBI grade.

PSM was employed to adjust for possible confounders between the groups and to reduce baseline differences between the two groups. Patients with ALBI scores of grades 1 were matched 1:1 with patients of grades 2 and 3, respectively. The results after matching are shown in Supplementary Material 2 below. The matched data were then examined further to explore the relation between ALBI and prognosis in HF patients with fewer confounding variables.

## Results

### Baseline characteristics

According to the ALBI grade, the 5845 eligible patients were classified into three groups (Fig. 1). As shown in Table 1, most of the covariates tested by the Kruskal-Wallis (KW) test exhibited significant between-group differences (*p* < 0.05). Compared to patients with ALBI grade 1, those with higher ALBI grades generally had elevated levels of INR, PT, ALT, ALP, AST, WBC, NtproBNP, heart rate (HR), SOFA score, lactate, creatinine, BUN, and total bilirubin (TBIL). At the same time, patients with higher ALBI grades had a higher probability of receiving MV and CRRT. Considering the substantial differences in sample sizes across the three groups, coupled with significant between-group differences in most covariates, this could lead to confounding bias and limit the generalizability of the study. Therefore, we performed propensity score matching to reduce confounding bias.

After performing propensity score matching (PSM), 516 matched pairs of patients were identified in ALBI class 1 versus class 2, and 339 matched pairs in ALBI class 1 versus class 3. Supplementary Material 2 summarizes the between-group differences in confounders after matching, which were assessed using the same statistical tests as before matching. There were no significant differences in the covariates after matching, except for INR and PT, which remained significantly different (*P* < 0.05), as determined by the appropriate statistical tests.

### ALBI grade for prognosis in patients with heart failure

The 30-, 90-, and 360-day all-cause mortality rates for heart failure (HF) patients were 24.17%, 33.46%, and 44.11%, respectively. Before PSM, the chi-square test revealed a significant difference in mortality among all three groups (Supplementary Material 3). Additionally, we explored the differences in mortality among the three groups after PSM (Supplementary Material 3). Analyses of ALBI classes 1 and 2 showed no significant difference in 30-day mortality between the two groups; however, the 90- and 360-day death rates for HF patients still differed significantly. In contrast, the mortality rate at grade 3 is still significantly higher than at grade 1 (Supplementary Material 3). In addition, multivariate Cox regression results are shown in Supplementary Material 4, with separate results before and after PSM. The analyses revealed that both ALBI grade 2 and grade 3 were independent risk factors for prognosis in patients with HF before PSM. However, after PSM, there was no longer a difference between ALBI grade 2 for mortality in patients with HF, whereas grade 3 remained an independent risk factor (*p* < 0.05). This indicates that the mortality rate for ALBI grade 3 was consistently and significantly higher than grade 1 across all models (Supplementary Material 4).

**Fig. 2:**
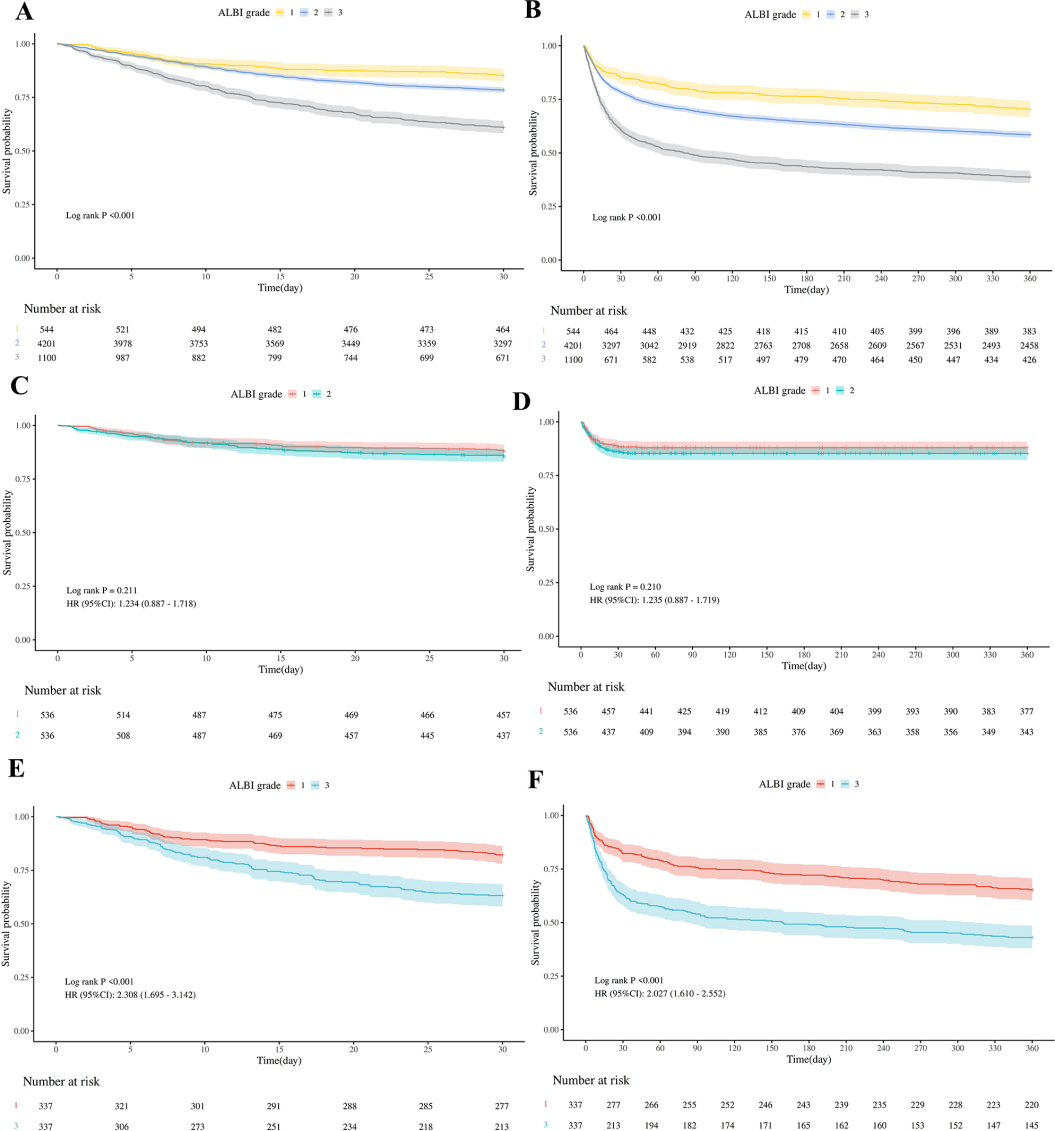
Kaplan-Meyer survival analysis curves for all-cause mortality: (**A**) *30-days all-cause mortality before PSM*; (**B**) 360-days all-cause mortality before PSM; (**C**) 30-days all-cause mortality after PSM with ALBI grade 1 paired with 2; (**D**) 360-days all-cause mortality after PSM with ALBI grade 1 paired with 2; (**E**) 30-days all-cause mortality after PSM with ALBI grade 1 paired with 3; (**F**) 360-days all-cause mortality after PSM with ALBI grade 1 paired with 3. ALBI grades 1, 2, and 3 are ≤ -2.60, − 2.60 to − 1.39, > -1.39 respectively. Abbreviations: ALBI, albumin-bilirubin; PSM, Propensity Score Matching

The Kaplan-Meier (K-M) curve results support the findings of the multivariate Cox regression analysis. The survival curves for the three ALBI grades before and after matching are shown in Fig. 2. Before PSM, it can be seen that there was a significant difference in the survival curves of ALBI grade 1, 2, and 3 groups (Log-rank *P* < 0.001), and the survival rate of patients with high grade (grade 3) was significantly lower than that of patients with low grade (grade 1) over time, a trend that was consistent across short- and long-term follow-ups. However, after the PSM, there was no difference in survival between ALBI grades 1 and 2 (*p* > 0.05) (Fig. 2). In contrast, after PSM, patients with ALBI grade 3 had a significantly higher risk of death than those with grade 1 in all time periods (*P* < 0.01), as indicated by a hazard ratio (HR > 2) that was more than twice as high as the risk of death for patients with grade 1 (Fig. 2). Supplementary Material 5 demonstrates the patient’s mid-term K-M survival curve.

**Fig. 3:**
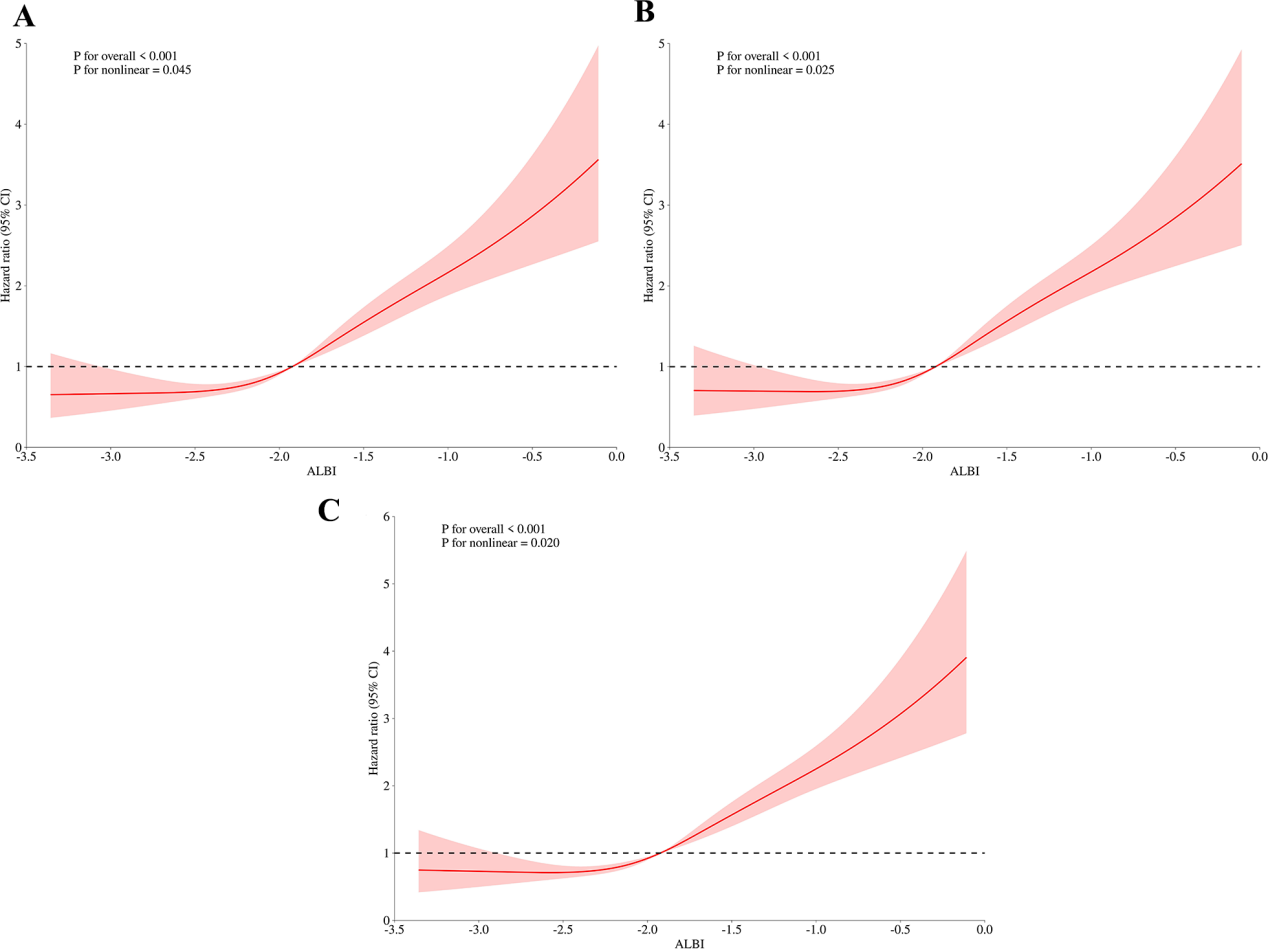
RCS analysis of ALBI score and mortality in patients with heart failure. Note: (**A**) RCS analysis of ALBI scores and 30-day mortality in patients admitted to hospitals; (**B**) 90-day (**C**) 360-day

In the RCS, our study variable ALBI was used as a rating, or continuous variable, which is different from the grade used as a categorical variable in other parts of this study. RCS demonstrated a statistically significant nonlinear association between ALBI score and 30-, 90-, and 360-day mortality in heart failure patients (P for nonlinear < 0.05) (Fig. 3). The reference value for all three mortality curves was determined to be -1.92. This suggests that all-cause mortality remains relatively stable at lower ALBI scores but increases sharply when the ALBI score approaches and exceeds − 1.92.

**Fig. 4:**
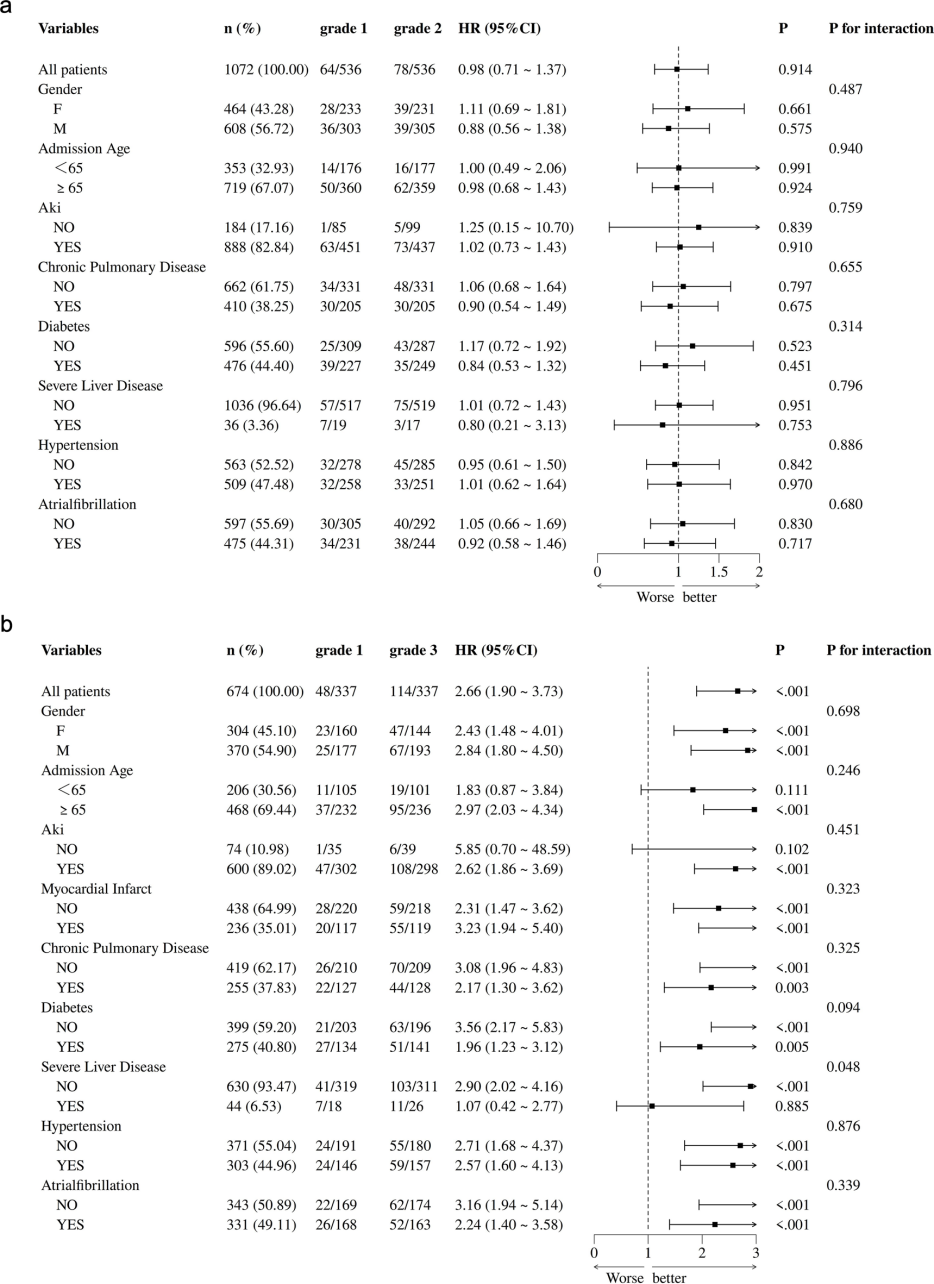
Effect of ALBI grading on 30-day mortality in different subgroups after PSM. Note: (**A**) Subgroup analyses of ALBI grade 1 paired with 2; (**B**) Subgroup analyses of ALBI grade 1 paired with 3

Subgroup analyses were performed for the two pairs after PSM, and the corresponding forest plots are presented in Fig. 4. None of the effect differences between these subgroups reached statistical significance, and no significant interactions were observed (*P* > 0.05), as shown in Fig. 4A. However, Fig. 4B demonstrates that there was a statistically significant difference (*P* < 0.05) between the matched ALBI grading (grade 1 vs. grade 3) in most subgroups, along with a significant interaction with severe hepatic impairment.

## Discussion

The predictive significance of ALBI grade in HF patients was examined in this study [20]. By analyzing 5,845 HF patients, we found that higher ALBI grades, especially grade 3 (> -1.39), were related to significantly increased all-cause mortality at 30, 90, and 360 days after admission. In addition, it is noteworthy that after adjusting for confounders by PSM, ALBI grade 3 remains an independent risk factor for HF patients, but grade 2 changed to a non-significant difference. Notably, patients with high ALBI grades received intensive care treatments (e.g., mechanical ventilation and CRRT) more frequently, which may reflect the severity of their disease. However, by multivariate adjustment, we confirmed that ALBI grade 3 predicted mortality independently of treatment differences, suggesting that liver impairment itself is a key driver of prognosis. The findings are supported by Kaplan–Meier (K–M) survival analyses, which indicated no significant difference in mortality between ALBI grades 1 and 2 following PSM (*P* > 0.05). In addition, the probability of death increased significantly when ALBI levels were above − 1.92, according to RCS.

Albumin and total bilirubin are used to calculate the ALBI score, which is mainly used for the assessment of patients with hepatic liver disease [12]. Hiraoka et al. [21] showed that the ALBI grade is better than the Child-Pugh classification in predicting survival and treatment outcomes in patients with hepatocellular carcinoma, especially in the group of patients with better liver function, and that the ALBI score is able to distinguish patients with different prognoses in a more accurate way. The ALBI score has also been used to assess several different illnesses lately. Gou et al. [15] discovered in a retrospective research study that high-grade ALBI was linked to a high death rate in sepsis patients. Through an analysis of ALBI grade and clinical results in acute renal injury patients, Yang et al. [17] found that the HR for in-hospital mortality was 2.34 (2.02, 2.70) and 5.17 (4.44, 6.02) for ALBI grades 2 and 3, respectively. After adjustment for covariates, the HR were 1.31 (1.11, 1.55) and 1.52 (1.24, 1.85), respectively, indicating that higher ALBI grades were strongly linked to a poorer prognosis for patients and that higher grades were correlated with a higher risk of death. In cardiac disease, however, previous studies have indicated an association of ALBI grade with death in HF patients, but none of them have performed a balance of confounders, and none of them have examined specific classifications [18, 22, 23].

Heart failure is a complex condition, often accompanied by multi-organ dysfunction, particularly liver impairment [24, 25]. The liver is crucial for metabolism and detoxification, and its dysfunction in HF patients can lead to worse outcomes, including a higher risk of mortality [26]. Several previous studies have found that raised bilirubin levels, as well as hypoalbuminemia, are associated with a higher risk of death in patients with HF [4, 27]. When compared to the MELD XI score, the ALBI score was a stronger indicator of liver function abnormalities and a better indicator of mortality in patients with HF, according to research by Matsue et al. [18]. This is consistent with our findings. The results of our study showed significant differences between patients with different ALBI scores, with 30-, 90-, and 360-day all-cause mortality rates being significantly higher in grade 3 (severe hepatic dysfunction) than in grade 1 (mild hepatic dysfunction) patients. This was confirmed by Han et al. [22]. However, his study was graded according to the triple classification and also did not have a propensity to match score, thus confounding factors were more influential.

Luo et al. [23] demonstrated that the ALBI score predicted both short-term and long-term mortality in HF patients and that its predictive value for short-term mortality was higher than for long-term mortality. However, in our study, with or without propensity score matching, the results showed that ALBI classification predicted long-term mortality more than short-term mortality, whereas this study found no statistically significant difference between ALBI classifications 1 and 2 in predicting mortality (*p* > 0.05). Such a reason could be the different conditions under which the patients were screened or the design of the experiment. These findings have significant clinical practice implications since they indicate that patients with severe liver function impairments and HF may need closer monitoring and more specialized treatment plans.

When the grouped sample sizes differ significantly, PSM is often used to match samples from different groupings, thereby reducing the impact of sample size imbalance [28]. In this study, we matched ALBI grade 1 with grades 2 and 3 for samples 1:1, respectively, followed by post-matching analyses. Table 3 displays the findings of our analyses. ALBI grades 2 and 3 are independent predictors of death among patients with pre-PFS HF, consistent with previous studies. However, this study differs from earlier studies in that the difference in all-cause mortality between ALBI grades 1 and 2 was not statistically significant (*P* > 0.05) during the 30-, 90-, and 360-day follow-up periods after application of PSM.

RCS analysis showed a nonlinear relationship between ALBI scores and death. Mortality rates remained stable at lower ALBI scores but increased sharply as the score approached − 1.92. This finding suggests that the ALBI score may exhibit a threshold effect, meaning that changes in mild to moderate impairments in liver function in patients with HF may have little effect on mortality (ALBI score < -1.92). At the same time, we should focus more on heart failure patients with ALBI scores close to -1.92, where timely improvement of liver function and slowing its deterioration can significantly reduce mortality. The association between ALBI score and mortality in HF patients has not been explicitly addressed in previous studies [22, 23, 29, 30]. At the same time, given the advantages of RCS in revealing complex nonlinear relationships, future studies should consider adopting this methodology, and prospective studies could be conducted to more fully and accurately assess the prognostic value of the ALBI score in HF patients.

This study used a different methodology than previous studies and also obtained different findings. However, this study does have a few limitations. Firstly, the present study is a single-center retrospective study, making it difficult to determine causality and only observe associations. Secondly, our study is limited by the lack of LVEF and device therapy (Implantable cardioverter-defibrillator / cardiac resynchronization therapy) data, inherent to ICU databases. Third, since the MIMIC database only provides all-cause mortality rates and not specific causes of death, further analysis on the causes of death is not possible. Finally, this study is a hypothesis-generating study that refers to a very specific situation of the patient with heart failure, which is the moment of admission to an ICU. The study did not consider the duration of heart failure progression or the patient’s functional class, both of which could have influenced the results. Therefore, it remains uncertain whether our findings can be generalized to a broader population of outpatient heart failure patients. Future studies should compare the ALBI score with these traditional risk models to assess its added value in clinical practice.

## Conclusion

ALBI grade 3 is an independent predictor of mortality in patients with HF. However, further studies will be necessary for confirmation of these findings.

## Electronic supplementary material

Below is the link to the electronic supplementary material.

Supplementary Material 1

Supplementary Material 2

Supplementary Material 3

Supplementary Material 4

Supplementary Material 5

## Data Availability

The data used in this study were obtained from the MIMIC-IV database (Medical Information Mart for Intensive Care), which is publicly available on the PhysioNet platform. Access to MIMIC-IV requires registration and completion of the Collaborative Institutional Training Initiative (CITI) program on human subject’s research. The dataset can be accessed at https://physionet.org/content/mimiciv. Researchers interested in using this data must apply for access following the guidelines provided by PhysioNet.

## Abbreviations

ALT: Alanine Aminotransferase
ALP: Alkaline Phosphatase
AST: Aspartate Aminotransferase
ALBI: Albumin-Bilirubin
AKI: Acute Kidney Injury
BUN: Blood Urea Nitrogen
CRRT: Continuous Renal Replacement Therapy
DBP: Diastolic Blood Pressure
HR: Heart Rate
RBC: Red Blood Cell
INR: International Normalized Ratio
MAP: Mean Arterial Pressure
PT: Prothrombin Time
SOFA: Sequential Organ Failure Assessment
SBP: Systolic Blood Pressure
TBil: Total Bilirubin
WBC: White Blood Cell
