# Supplementary material for: Association of albumin-bilirubin grade with short- and long-term mortality in patients with heart failure: a cohort study using restricted cubic splines and propensity score matching
Source: BMC Cardiovasc Disord. 2025 Apr 23;25:307. doi: 10.1186/s12872-025-04760-2 (PMC12016267; doi:10.1186/s12872-025-04760-2)
Supplement: Supplementary file 5 — Supplementary Material 5 [file 12872_2025_4760_MOESM5_ESM.docx]

**Supplementary Table 1** The ICD-9 and ICD-10 codes for identifying heart failure.

|  | **ICD-9 codes** | **ICD-10 codes** |
| --- | --- | --- |
| Heart failure | 4280 4281 4289 39891 40291 40491 40493 42820 42821 42822 42823 42830 42831 42832 42833 42840 42841 42842 42843 | I0981 I110 I130 I132 I5020  I5021 I5022 I5023 I5030 I5031  I5032 I5033 I5040 I5041 I5042  I5043 I50810 I50811 I5082  I5084 I509 I97130 I97131 |

**
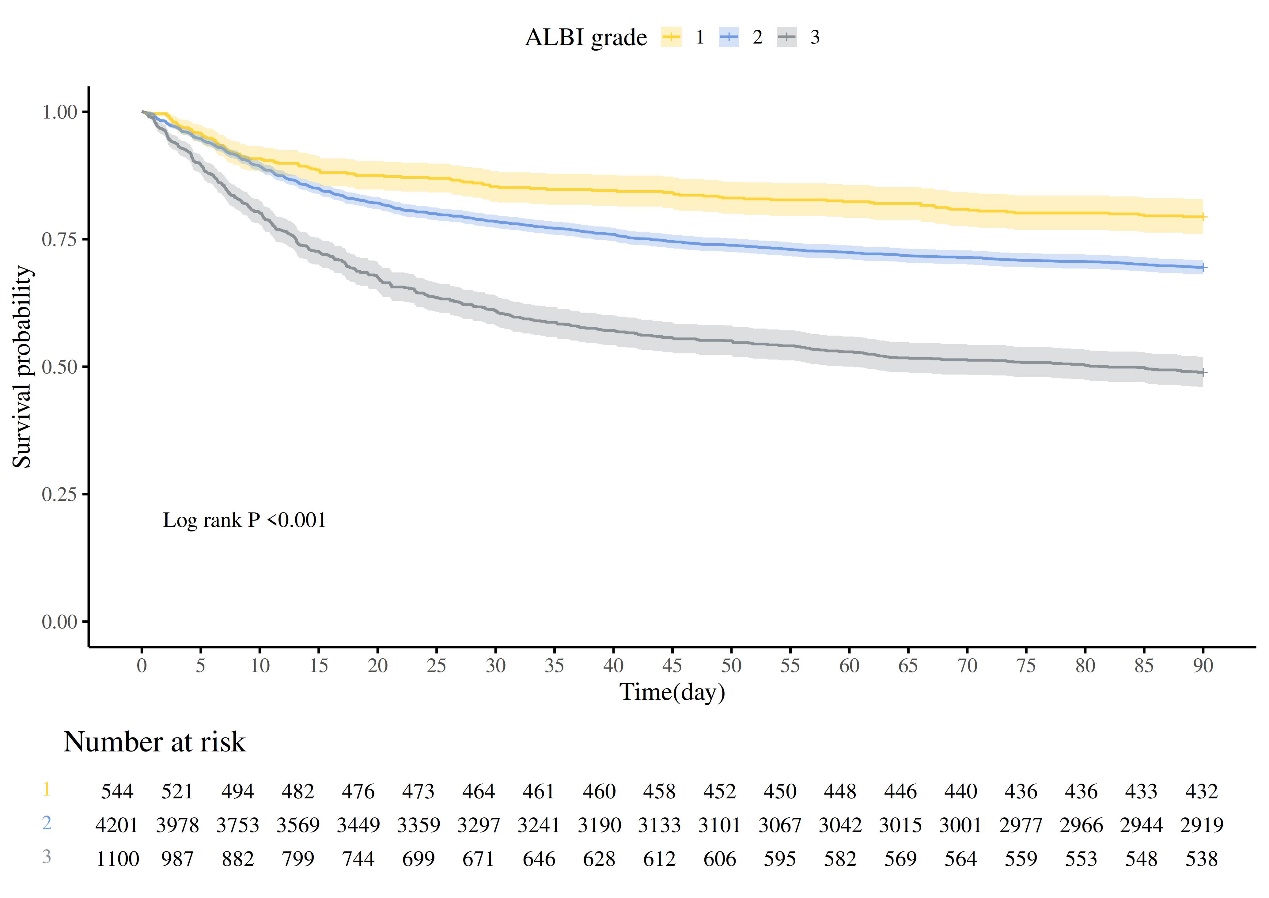
**

**Supplementary Fig. 1A** Kaplan-Meier curves for all-cause mortality according to ALBI grade in heart failure patients: *90-days all-cause mortality before PSM*;

**
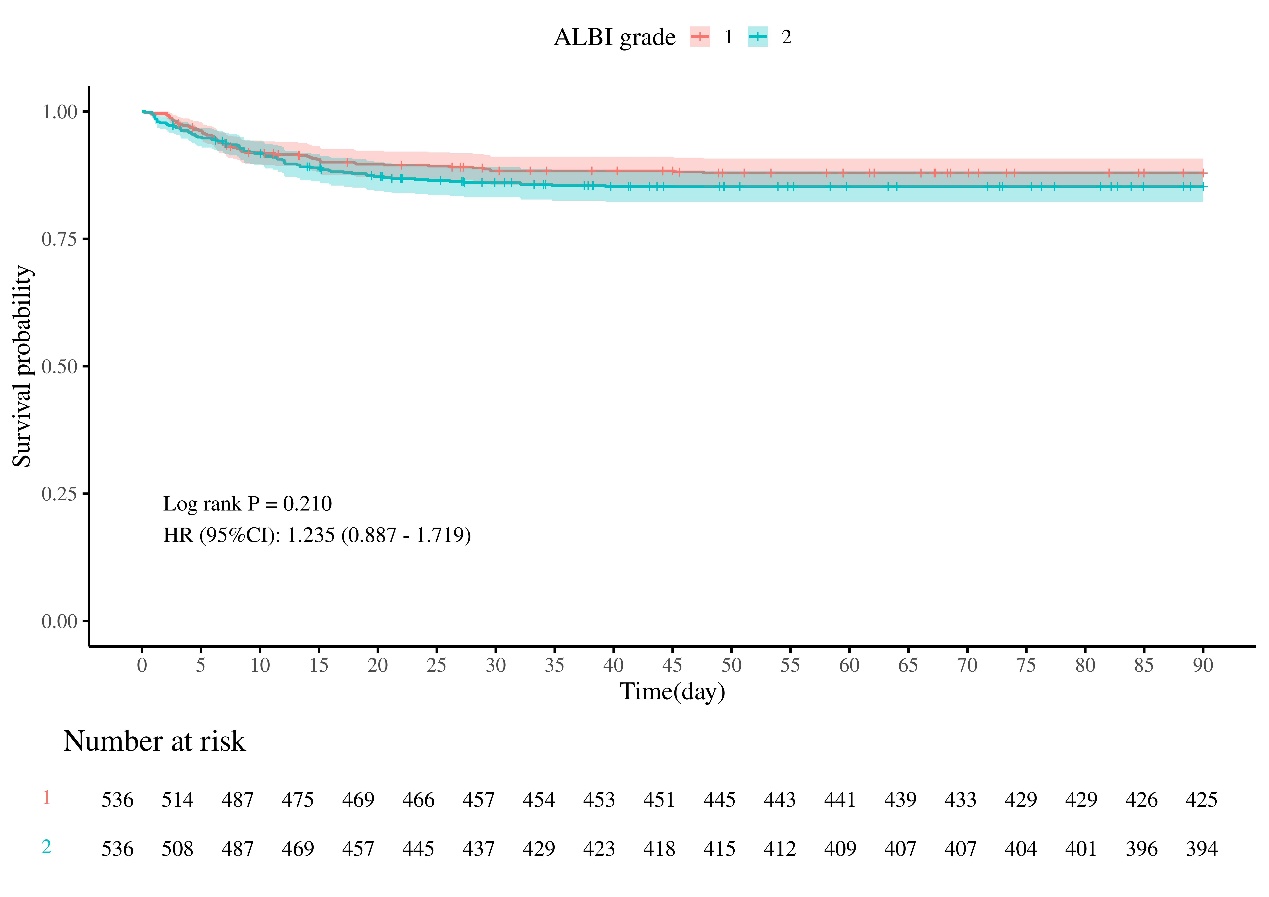
**

**Supplementary Fig. 1B** Kaplan-Meier curves for all-cause mortality according to ALBI grade in heart failure patients: 90-days all-cause mortality after PSM with ALBI grade 1 paired with 2;

**
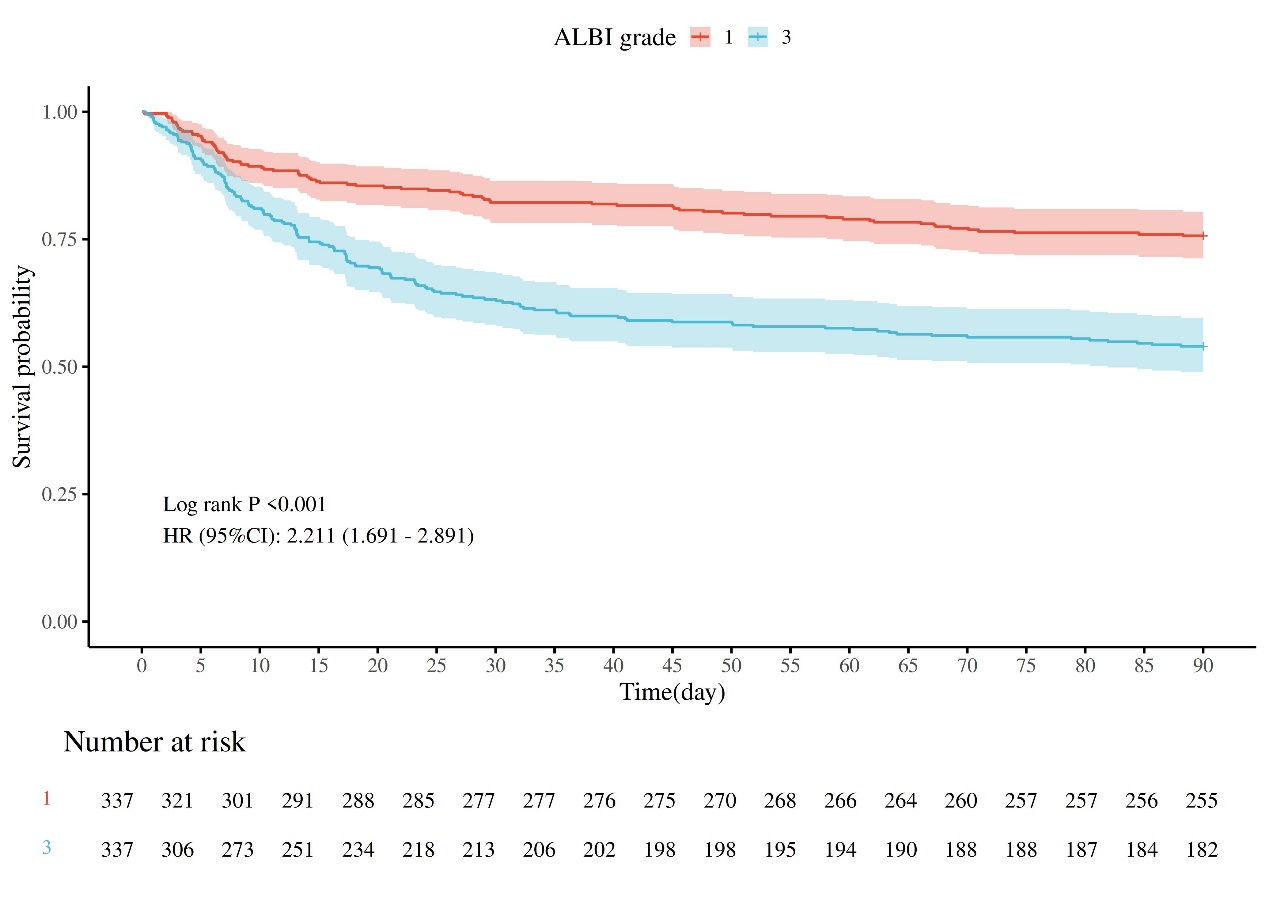
**

**Supplementary Fig. 1C** Kaplan-Meier curves for all-cause mortality according to ALBI grade in heart failure patients: 90-days all-cause mortality after PSM with ALBI grade 1 paired with 3;
